# Synergistic Effect of Simultaneous versus Sequential Combined Treatment of Histone Deacetylase Inhibitor Valproic Acid with Etoposide on Melanoma Cells

**DOI:** 10.3390/ijms221810029

**Published:** 2021-09-17

**Authors:** Yueh-Ming Shyu, Lawrence Yu-Min Liu, Yung-Jen Chuang

**Affiliations:** 1 Institute of Bioinformatics and Structural Biology, National Tsing Hua University, Hsinchu 30013, Taiwan; yuehmingshyu@gmail.com; 2 Department of Medical Science, National Tsing Hua University, Hsinchu 30013, Taiwan; 3 Department of Internal Medicine, Division of Cardiology, Hsinchu MacKay Memorial Hospital, Hsinchu 30071, Taiwan; 4 Department of Medicine, MacKay Medical College, New Taipei City 25245, Taiwan

**Keywords:** synergistic effect, melanoma, histone deacetylase inhibitor (HDACi), combination therapy, drug sequential order

## Abstract

Melanoma is the most lethal form of skin cancer, which is intrinsically resistant to conventional chemotherapy. Combination therapy has been developed to overcome this challenge and show synergistic anticancer effects on melanoma. Notably, the histone deacetylase inhibitor, valproic acid (VPA), has been indicated as a potential sensitizer of chemotherapy drugs on various metastatic cancers, including advanced melanoma. In this study, we explored whether VPA could serve as an effective sensitizer of chemotherapy drug etoposide (ETO) on B16-F10 and SK-MEL-2-Luc melanoma cell lines in response to drug-induced DNA damages. Our results demonstrated that the VPA-ETO simultaneous combined treatment and ETO pretreated sequential combined treatment generated higher inhibitory effectivities than the individual treatment of each drug. We found the VPA-ETO simultaneous combined treatment contributed to the synergistic inhibitory effect by the augmented DNA double-strand breaks, accompanied by a compromised homologous recombination activity. In comparison, the ETO pretreated sequential combined treatment led to synergistic inhibitory effect via enhanced apoptosis. Surprisingly, the enhanced homologous recombination activity and G2/M phase arrest resulted in the antagonistic effect in both cells under VPA pretreated sequential combined treatment. In summary, our findings suggested that sequential order and effective dose of drug administration in VPA-ETO combination therapy could induce different cellular responses in melanoma cells. Such understanding might help potentiate the effectiveness of melanoma treatment and highlight the importance of sequential order and effective dose in combination therapy.

## 1. Introduction

Melanoma is an aggressive cancer type with high metastatic potential. Ten percent of melanoma patients are already advanced stage at diagnosis [1]. When tumor cells disseminating to the lungs and liver, radiation therapy is ineffective in controlling the cancer progression [2]. To combat advanced melanoma, systemic therapies such as chemotherapy, targeted therapy, and immunotherapy are preferred treatment options. Unfortunately, there are limitations to these therapies, including the development of resistance or second mutations [3]. Chemotherapy is still the major systemic therapy for many patients due to affordability constraints. Hence, there remains a need for chemotherapy-based strategies for advanced melanoma.

In oncology, two or more anticancer drugs are usually used simultaneously or sequentially to improve the effectiveness. The enhanced efficacy of combination therapy mostly stems from the elimination of cancer cells that display partial response to a single anticancer agent alone [4]. Furthermore, combination therapy can sometimes demonstrate a synergistic effect, which is a nonlinear cumulative effect of two or more active ingredients with sequential or supplemental activities [5]. Combination therapy can be categorized into sequential or simultaneous combined treatments, which means the single therapeutic agents being administered separately or in a concurrent, fixed-dose combination [6]. Interestingly, Valentini et al. has reported that sequential orders of histone deacetylase inhibitor valproic acid (VPA) with chemotherapeutic agents etoposide (ETO) or cisplatin administration can contribute to different extents of melanoma cell inhibition in vitro [7]. This suggested that the sequential drug order might affect the inhibitory effect on cancer cells. However, there is no information on drug synergism and the underlying action mechanism of the VPA-ETO combined treatment [7]. Since the VPA-ETO combination has the potential to eliminate melanoma effectively, we chose to use this drug treatment scheme to answer questions on planning the administration scheme of medication.

VPA has been shown to provokes strong apoptotic effects and increases the expression levels of p21 and other cell cycle regulators to induce G1 phase arrest in melanoma cells [8]. VPA thus sensitized other melanoma therapies, such as chemotherapy and radiation therapy, from both in vitro and in vivo studies [7,9,10]. On the other hand, the topoisomerase II inhibitor etoposide (ETO) exerted enhanced cytotoxicity and induced caspase-dependent mitochondrial apoptosis in melanoma cells [11]. It shall be noted that ETO has already been shown to enhance cytotoxicity and apoptosis when combined with VPA in several cancers [7,12,13]. However, a previous phase I/II clinical trial that combined VPA with chemo-immunotherapy on advanced melanoma patients did not perform better than standard therapy and even resulted in more adverse effects [14]. Such contradictive findings between basic research and clinical trial have gained our attention. We thus aimed to investigate the potential synergistic effect of VPA and ETO on inhibiting melanoma and to examine whether the sequential order of VPA and ETO could be a key factor in exerting the synergistic anticancer effect. We would also explore how DNA damage responses, cell cycle arrest, and apoptosis might be altered in melanoma cells under different treatment schemes of VPA and ETO.

## 2. Results

### 2.1. Analysis of Dose-Response Curves under Single Treatments of VPA or ETO on B16-F10 and SK-MEL-2-Luc Melanoma Cells

We first evaluated the efficacies of single treatments of VPA or ETO on murine melanoma cell line B16-F10 and human melanoma cell line SK-MEL-2-Luc to determine the IC_50_ values of each drug. Both B16-F10 and SK-MEL-2-Luc cells were treated with the specified drug for 72 h, and the cell proliferative activities were then evaluated by cell viability assay.

**Figure 1:**
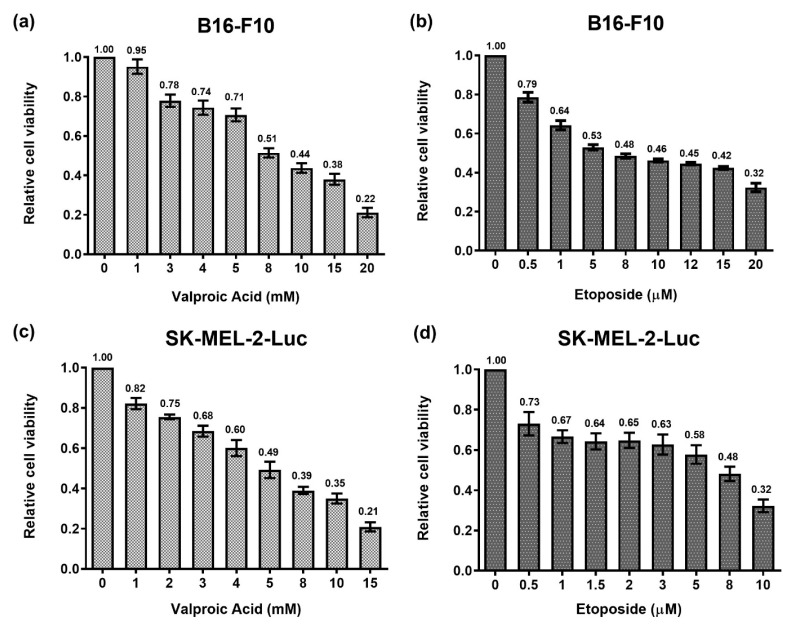
Dose-response analysis of VPA or ETO single treatment on melanoma B16-F10 and SK-MEL-2-Luc cells. The relative cell viability of B16-F10 cells was evaluated through cell viability assay after 72 h of single-drug treatments with either (**a**) VPA or (**b**) ETO. The relative cell viability of SK-MEL-2-Luc cells was evaluated likewise in response to (**c**) VPA or (**d**) ETO. The data are shown as means ± standard error of the mean (SEM) of independent experiments in triplicate.

**Figure 2:**
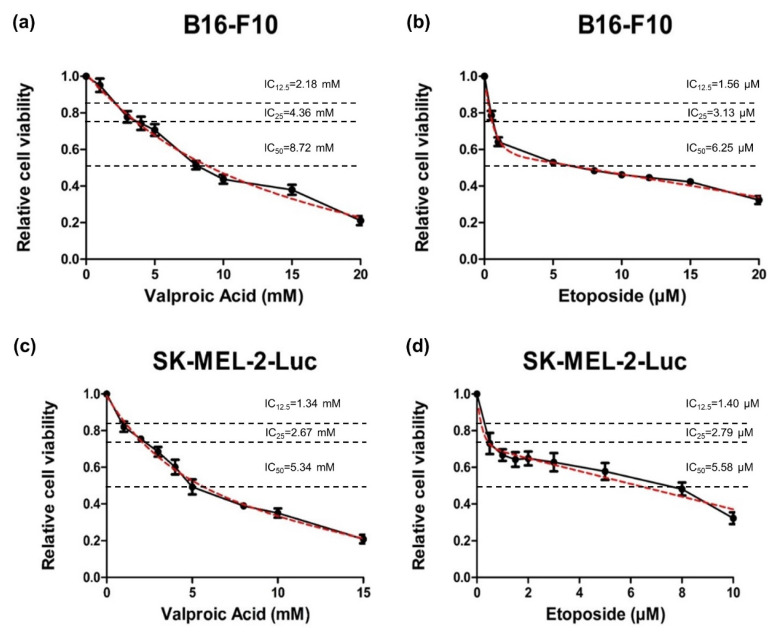
Curve fitting analysis to determine the effective inhibitory concentrations of VPA and ETO. To obtain IC_50_s of VPA and ETO for subsequent experiments, exponential two-phase decay model (marked as dashed red line) was fitted to the dose-response curves (solid black line, representing data shown in Figure 1) of single treatment of (**a**) VPA (R^2^ = 0.9531), or (**b**) ETO (R^2^ = 0.9841) on B16-F10 cells; and single treatment of (**c**) VPA (R^2^ = 0.9571), or (**d**) ETO (R^2^ = 0.8415) on SK-MEL-2-Luc cells. The dose-response fitting curves were used to calculate the IC_12.5_, IC_25_ and IC_50_ as follow: Concentration of VPA in B16-F10 cells: IC_12.5_ = 2.18 mM, IC_25_ = 4.36 mM, and IC_50_ = 8.72 mM; Concentration of ETO in B16-F10 cells: IC_12.5_ = 1.56 μM, IC_25_ = 3.13 μM, and IC_50_ = 6.25 μM. Concentration of VPA in SK-MEL-2-Luc cells: IC_12.5_ = 1.34 mM, IC_25_ = 2.67 mM, and IC_50_ = 5.34 mM; Concentration of ETO in SK-MEL-2-Luc cells: IC_12.5_ = 1.40 μM, IC_25_ = 2.79 μM, and IC_50_ = 5.58 μM. The analysis was carried out by using GraphPad Prism 8 (GraphPad software, San Diego, CA, USA). The data are shown as means ± SEM of independent experiments in triplicate.

The results demonstrated that the single treatments of VPA or ETO had dose-dependent inhibitory effects on B16-F10 and SK-MEL-2-Luc cells (Figure 1). The exponential two-phase decay model [15] was then used to estimate the corresponding cell inhibition parameters of B16-F10 and SK-MEL-2-Luc cells under VPA or ETO single treatment at the specified drug concentrations (Figure 2). The IC_50_ values of VPA and ETO on B16-F10 cells were determined to be 8.72 mM and 6.25 μM, respectively (Table 1). Notably, the IC_50_ values of VPA and ETO were lower on SK-MEL-2-Luc cells at 5.34 mM and 5.58 μM, respectively (Table 1). These data indicated that SK-MEL-2-Luc cells were more sensitive to VPA and ETO than B16-F10 cells.

### 2.2. Simultaneous and Sequential Combined Treatments of VPA and ETO Contributed to Synergistic Inhibitory Effects or Antagonistic Effect

**Figure 3:**
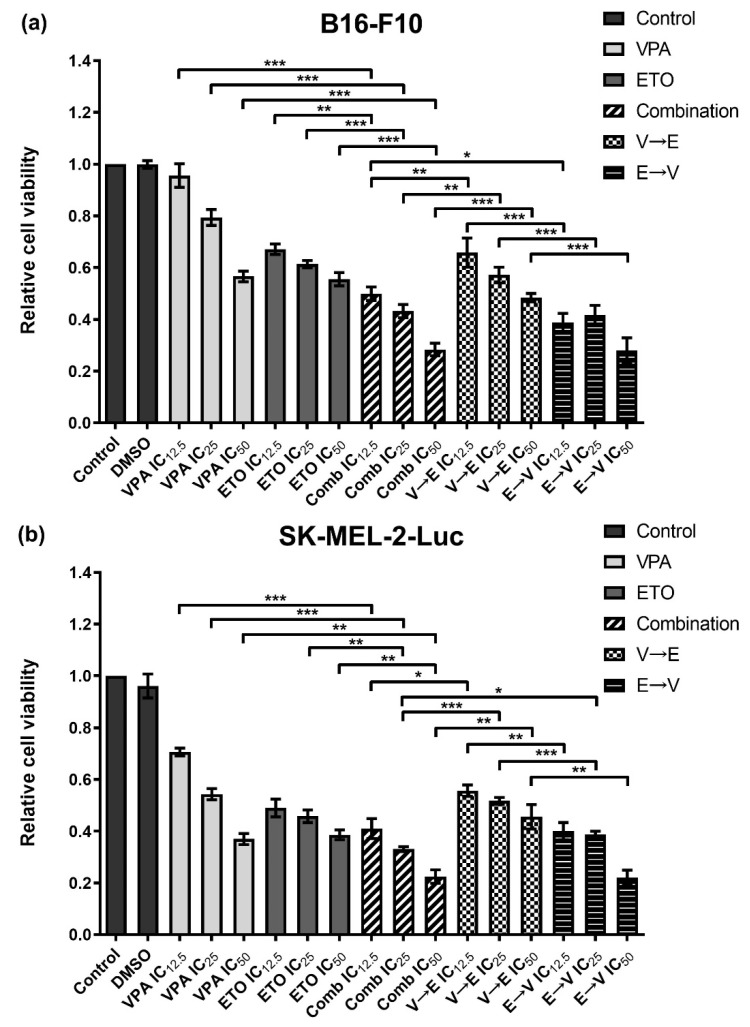
Inhibition effects of single versus combination treatments of VPA and ETO on melanoma cells. The relative cell viability was evaluated after 72 h single, simultaneous, or sequential combined treatments of VPA and/or ETO on (**a**) B16-F10 and (**b**) SK-MEL-2-Luc cells at the corresponding IC_12.5_, IC_25_, and IC_50_ determined in Figure 2. The data are shown as means ± SEM of independent experiments in triplicate. (* *p* < 0.05; ** *p* < 0.01; *** *p* < 0.001).

After determining the IC_50_ values of VPA and ETO, we investigated the cell inhibition effects under single treatments, simultaneous and sequential combined treatments of VPA and ETO, at the concentrations of IC_12.5_, IC_25_, and IC_50_ on B16-F10 and SK-MEL-2-Luc cells (Figure 3).

In Figure 3, the simultaneous combined treatment resulted in much lower cell viabilities than single-drug treatment on both cell lines. These results showed that the simultaneous combined treatment of VPA and ETO exhibited enhanced inhibitory effects on both cell lines compared to single treatments of VPA or ETO. We also found that the drug orders in sequential combined treatments of VPA and ETO contributed to different inhibitory effects on B16-F10 and SK-MEL-2-Luc cells. As shown in Figure 3, VPA→ETO sequential treatment resulted in much higher cell viabilities of B16-F10 and SK-MEL-2-Luc cells than the other combination conditions. These data implied that enhanced inhibitory effect appeared under two conditions: (1) simultaneous combined treatment of VPA and ETO, and (2) ETO→VPA sequential combined treatment. Therefore, these data suggested that the specific drug effect could be altered in different sequential orders.

To better evaluate the drug synergism, we adapted Chou and Talalay’s combination index (CI) method [16,17] to quantitate the degrees of the synergistic outcome. Categorization of different degrees of synergism and the corresponding ranges of CI value are described in Supplementary Materials Table S1 [17].

**Figure 4:**
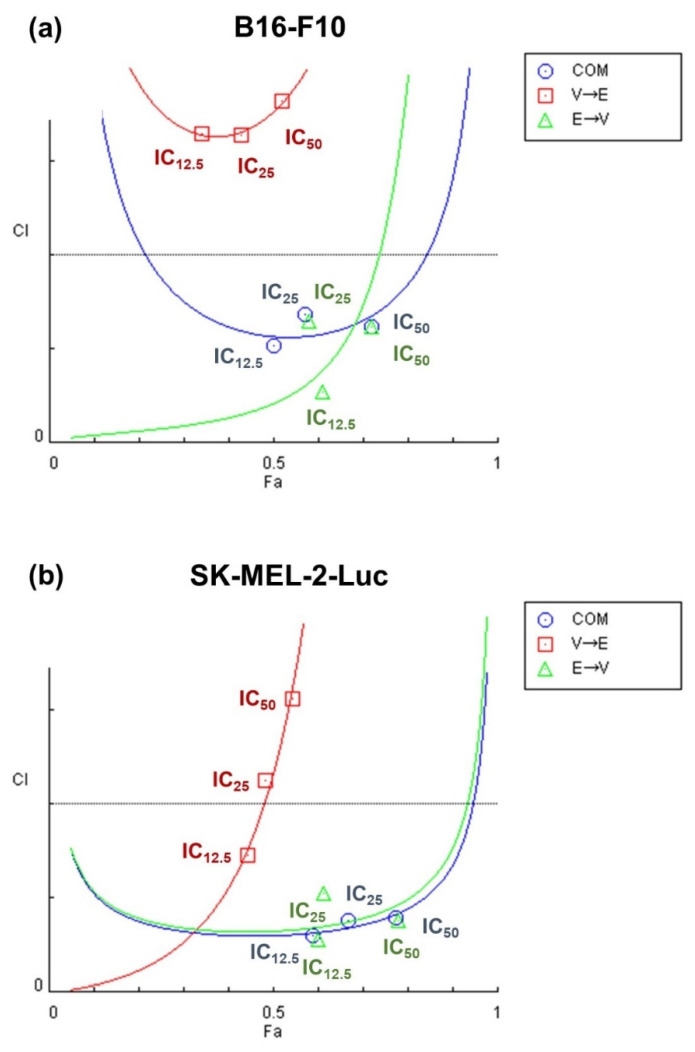
Fa-CI plot analysis of the synergistic and antagonistic effects of different VPA and ETO treatment schemes on inhibiting melanoma cells. The combination index (CI) values for simultaneous versus sequential combined treatments of VPA and ETO treatment schemes on (**a**) B16-F10 and (**b**) SK-MEL-2-Luc cells were calculated based on Chou and Talalay’s method via CompuSyn analysis software, 2005 Edition (ComboSyn, Paramus, NJ, USA). This analysis evaluates the effectiveness of combination treatment under the specified order of drug administration. In the Fa-CI plot, fractions of affected cells (Fa) represent the dead cells after drug treatments. Circle, square, and triangle symbols represent the CI values of each Fa under the specified treatment scheme with different orders of drug administration. The range of Fa is from 0 (no inhibition) to 1 (complete inhibition). The CI values are quantitative grading of synergism (CI < 1), additive (CI = 1), and antagonism (CI > 1). The IC_12.5_, IC_25,_ and IC_50_ values are listed in Figure 2. IC_50_ values are summarized in Table 1.

As shown in Figure 4, we observed synergism under simultaneous and ETO→VPA sequential treatments in both cell lines at the concentrations of IC_12.5_, IC_25_, and IC_50_. Surprisingly, antagonism was observed under VPA→ETO sequential treatment at three concentrations in B16-F10 cells (Figure 4a) and the concentrations of IC_25_ and IC_50_ in SK-MEL-2-Luc cells (Figure 4b).

**Figure 5:**
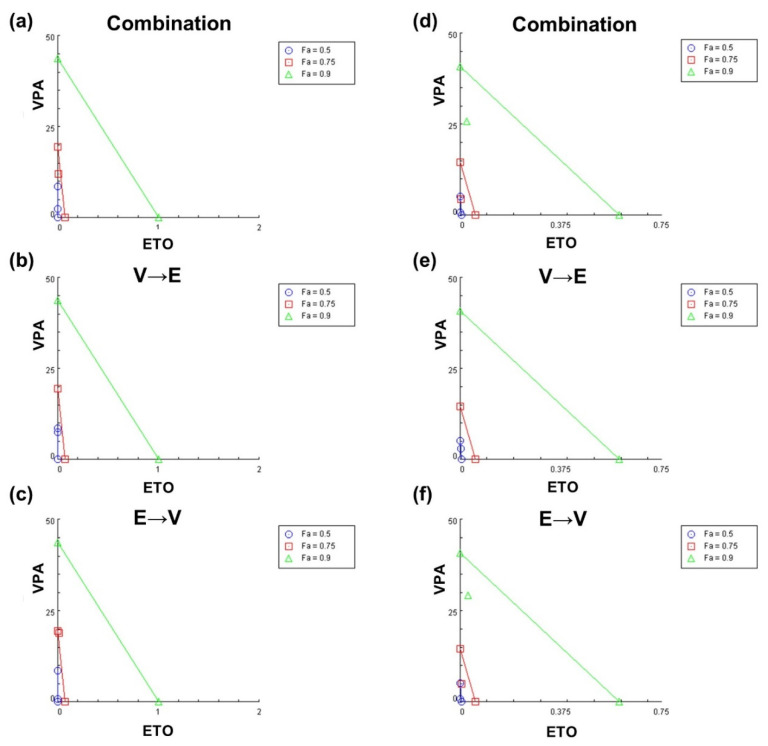
Isobologram analysis of the synergistic and antagonistic effects of different VPA and ETO treatment schemes. The isobolograms analysis revealed the degrees of effectiveness of the specified treatment scheme on inhibiting B16-F10 and SK-MEL-2-Luc cells. Specifically, (**a**) simultaneous combined treatment of VPA and ETO, (**b**) sequential combination with VPA pretreatment, and (**c**) sequential combination with ETO pretreatment in B16-F10 cells; (**d**) simultaneous combined treatment of VPA and ETO, (**e**) sequential combination with VPA pretreatment, and (**f**) sequential combination with ETO pretreatment in SK-MEL-2-Luc cells. The individual doses of VPA and ETO that attained 50% (Fa = 0.5), 75% (Fa = 0.75), and 90% (Fa = 0.9) inhibitory effects (i.e., effective dose, ED) are designated as blue lines, red lines, and green lines, respectively. The CI values at ED_50_, ED_75_, and ED_90_ are indicated as blue circle, red square, and green triangle symbols, respectively. Symbols above the line, on the line, or below the line represent antagonistic, additive, or synergistic effects, respectively.

To evaluate both synergistic and antagonistic interactions of drug treatment schemes, we generated isobolograms to quantify the drug synergism at different effective doses (EDs) (Figure 5) as previously described [16,17]. The CI values under different drug sequential orders at ED_50_, ED_75_, and ED_90_ are summarized in Supplementary Materials Table S2. The results in Figure 5 indicated that while synergistic effects were observed under certain drug combination conditions, the outcome could invert to antagonistic effect as the effective dose increased. Moreover, these data also revealed that synergism might only exist within a limited range of effective doses of drug combinations.

### 2.3. ETO Dominated the Induction of DNA Double-Strand Breaks (DSBs) under ETO→VPA Sequential Combined Treatment

Since sequential order and effective dose of VPA and ETO administration could influence the inhibitory effects, we next investigated the molecular mechanisms underlying the drug actions of each administration scheme.

**Figure 6:**
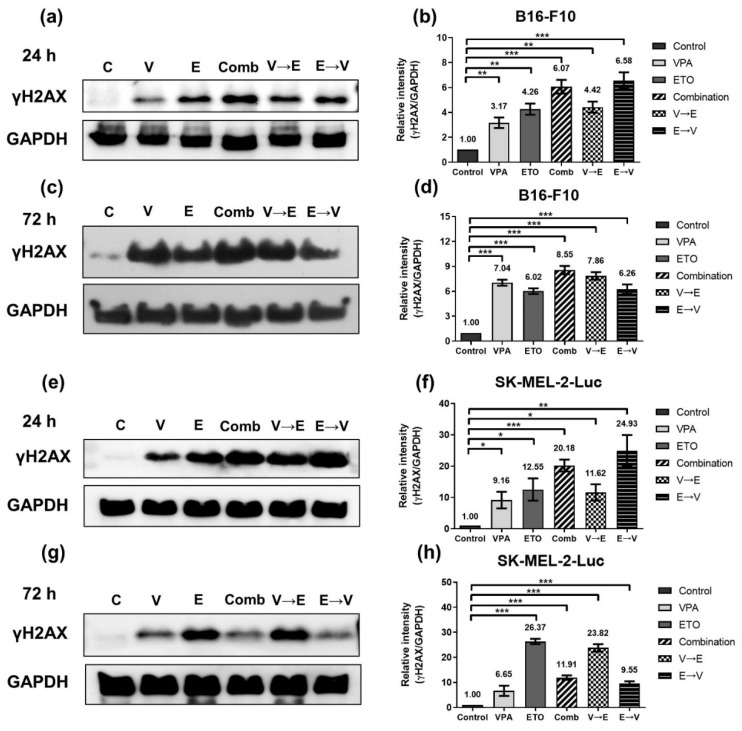
DNA damage induced by single, simultaneous, and sequential combined treatments of VPA and ETO in melanoma cells. Western blot analysis illustrated the DNA damage induced γH2AX expression after (**a**) 24 h (**c**) 72 h single, simultaneous, and sequential combined treatments of VPA and ETO in B16-F10 cells. (**b**,**d**) Quantitative analysis of (**a**,**c**) was obtained by using Image J software 1.52a (National Institutes of Health, Bethesda, MD, USA) and normalized with the no treatment control. The analysis was repeated in SK-MEL-2-Luc cells. Western blot data of (**e**) 24 h and (**g**) 72 h was shown, and the corresponding quantitative analysis was shown in (**f**,**h**), respectively. GAPDH was used as the control of sample loading. Relative intensities of γH2AX to GAPDH expressions were normalized with the control. The data are shown as means ± SEM of independent experiments in triplicate. (* *p* < 0.05; ** *p* < 0.01; *** *p* < 0.001). The drug dosages were referred to IC_50_ values as shown in Figure 2 and Table 1.

We first investigated whether the level of DNA DSBs might associate with the synergistic inhibitory effects. Western blot was used to examine the protein expression of γH2AX, which is the standard marker to report the extent of DNA DSBs (Figure 6) [18].

Compared with the control, the results demonstrated that the expression of γH2AX increased in B16-F10 cells under ETO single treatment and VPA→ETO sequential treatment for 24 h (Figure 6a,b). After drug treatments for 72 h, the expression levels of γH2AX kept increasing under VPA or ETO single treatments, simultaneous and VPA→ETO sequential treatment (Figure 6c,d). However, the expression of γH2AX in B16-F10 cells under ETO→VPA sequential treatment slightly decreased from 24 to 72 h (i.e., 6.58- to 6.26-fold). In SK-MEL-2-Luc cells, the expression of γH2AX increased under ETO single treatment and VPA→ETO sequential treatment for 24 h (Figure 6e,f). Surprisingly, the expression levels of γH2AX under ETO single treatment and VPA→ETO sequential treatment significantly increased while the expression levels of γH2AX sharply decreased under the other drug administration conditions. (Figure 6g,h). These results suggested that ETO dominated the expression levels of γH2AX under ETO→VPA sequential treatment.

### 2.4. DNA DSBs Induced by VPA Pretreated Sequential Combined Treatment Were Predominantly Repaired via Homologous Recombination

To investigate whether the difference in DNA repair could influence the inhibitory effects under different sequential orders of VPA and ETO administration, we analyzed DNA repair markers involved in the homologous recombination (HR) pathway or non-homologous end joining (NHEJ) pathway.

**Figure 7:**
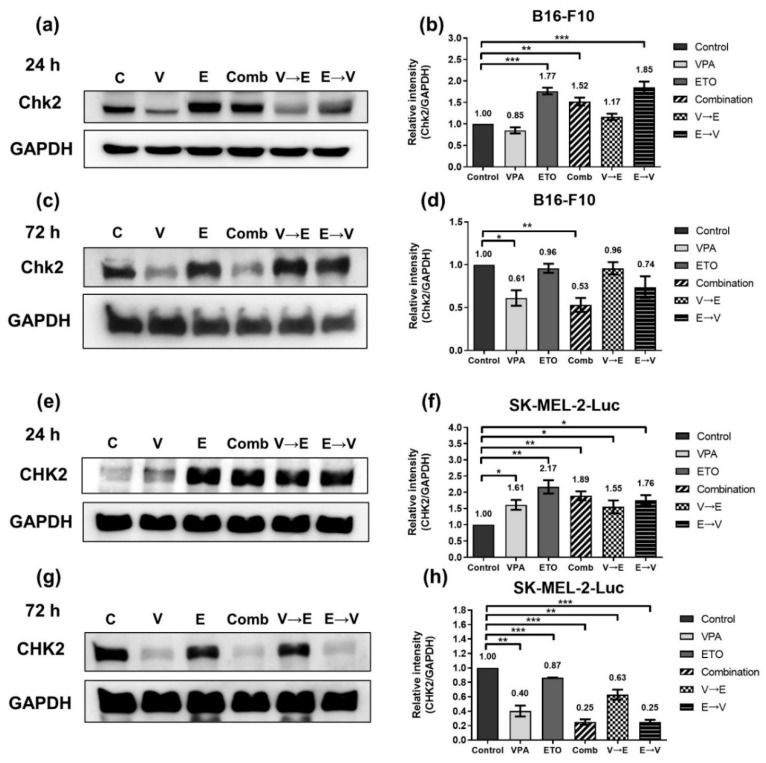
Expression of homologous recombination-related protein Chk2/CHK2, under single, simultaneous, and sequential combined treatments of VPA and ETO in melanoma cells. Western blot analysis illustrated the Chk2/CHK2 protein expression after (**a**) 24 h (**c**) 72 h single, simultaneous, and sequential combined treatments of VPA and ETO in B16-F10 cells. (**b**,**d**) was quantitative analysis of (**a**,**c**), respectively, which was obtained by using Image J software 1.52a (National Institutes of Health, Bethesda, MD, USA). The analysis was repeated in SK-MEL-2-Luc cells. Western blot data of (**e**) 24 h and (**g**) 72 h was shown, and the corresponding quantitative analysis was shown in (**f**,**h**), respectively. GAPDH was used as the control of sample loading. Relative intensities of Chk2/CHK2 to GAPDH expressions were normalized with the control. The data are shown as means ± SEM of independent experiments in triplicate. (* *p* < 0.05; ** *p* < 0.01; *** *p* < 0.001). The drug dosages were referred to IC_50_ values as shown in Figure 2 and Table 1.

As shown in Figure 7a,b, the HR-related DNA damage signal transducer Chk2 in B16-F10 cells was slightly down-regulated under VPA single treatment, while the expressions of Chk2 increased in conditions with ETO administration for 24 h. These results indicated that ETO dominated the induction of Chk2 protein expression levels. After 72 h drug administrations, similar expression levels of Chk2 were observed under either ETO single treatment or VPA→ETO sequential treatment compared to the control (Figure 7c,d). These results indicated that VPA single treatment might down-regulate Chk2 expression, while the administration of ETO following VPA might counteract Chk2 down-regulation.

As shown in Figure 7e,f, the expression levels of CHK2 under the specified treatment schemes were repeated in SK-MEL-2-Luc cells. The results also demonstrated that ETO dominated the expression level of CHK2. It is noted that the variation range of CHK2 expression levels in SK-MEL-2-Luc cells was broader than it in B16-F10 cells.

**Figure 8:**
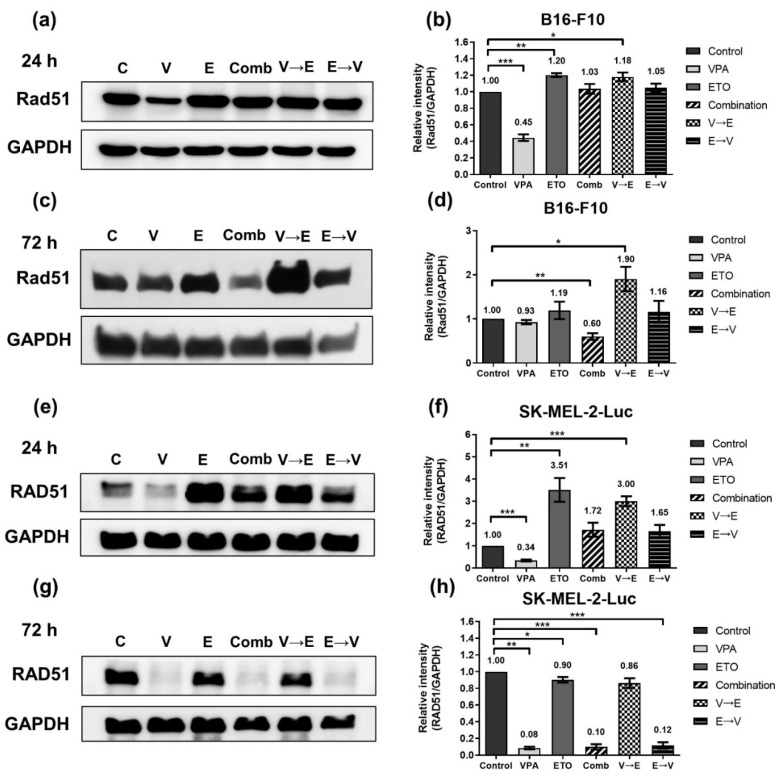
Expression of homologous recombination-related protein Rad51/RAD51, under single, simultaneous, and sequential combined treatments of VPA and ETO in melanoma cells. Western blot analysis illustrated the Rad51/RAD51 protein expression after (**a**) 24 h (**c**) 72 h single, simultaneous, and sequential combined treatments of VPA and ETO in B16-F10 cells. (**b**,**d**) was quantitative analysis of (**a**,**c**), respectively, which was obtained by using Image J software 1.52a (National Institutes of Health, Bethesda, MD, USA). The analysis was repeated in SK-MEL-2-Luc cells. Western blot data of (**e**) 24 h and (**g**) 72 h was shown, and the corresponding quantitative analysis was shown in (**f**,**h**), respectively. GAPDH was used as the control of sample loading. Relative intensities of Rad51/RAD51 to GAPDH expressions were normalized with the control. The data are shown as means ± SEM of independent experiments in triplicate. (* *p* < 0.05; ** *p* < 0.01; *** *p* < 0.001). The drug dosages were referred to IC_50_ values as shown in Figure 2 and Table 1.

**Figure 9:**
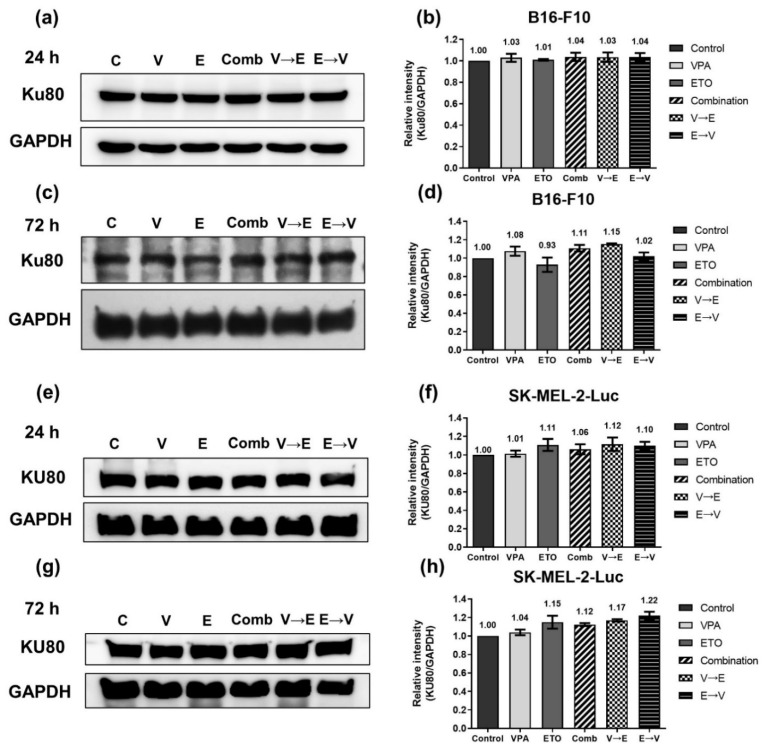
Expression of non-homologous end joining (NHEJ) related protein Ku80/KU80, under single, simultaneous, and sequential combined treatments of VPA and ETO in melanoma cells. Western blot analysis illustrated the Ku80/KU80 protein expression after (**a**) 24 h (**c**) 72 h single, simultaneous, and sequential combined treatments of VPA and ETO in B16-F10 cells. (**b**,**d**) was quantitative analysis of (**a**,**c**), respectively, which was obtained by using Image J software 1.52a (National Institutes of Health, Bethesda, MD, USA). The analysis was repeated in SK-MEL-2-Luc cells. Western blot data of (**e**) 24 h and (**g**) 72 h was shown, and the corresponding quantitative analysis was shown in (**f**,**h**), respectively. GAPDH was used as the control of sample loading. Relative intensities of Ku80/KU80 to GAPDH expressions were normalized with the control. The data are shown as means ± SEM of independent experiments in triplicate. The drug dosages were referred to IC_50_ values as shown in Figure 2 and Table 1.

**Figure 10:**
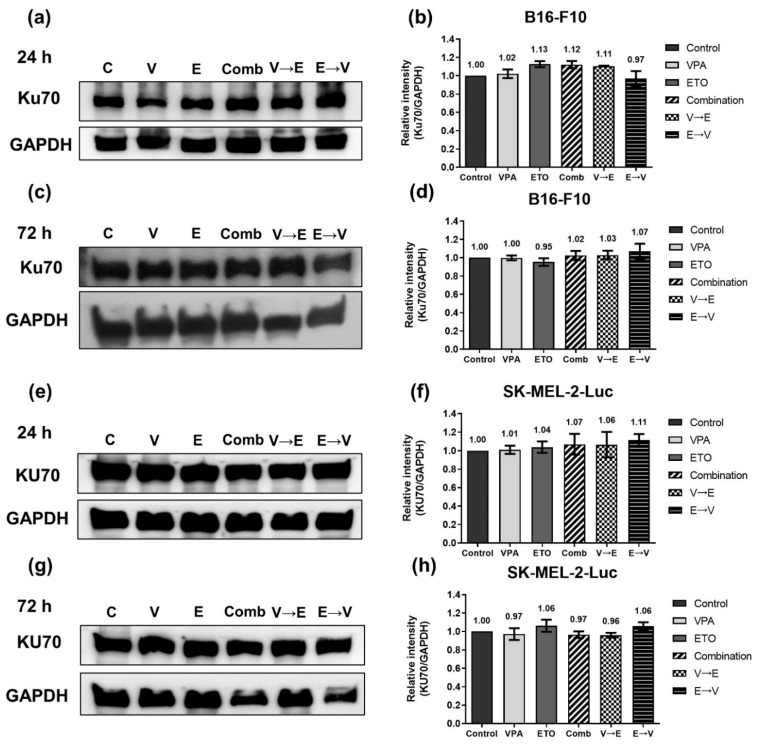
Expression of non-homologous end joining (NHEJ) related protein Ku70/KU70, under single, simultaneous, and sequential combined treatments of VPA and ETO in melanoma cells. Western blot analysis illustrated the Ku70/KU70 protein expression after (**a**) 24 h (**c**) 72 h single, simultaneous, and sequential combined treatments of VPA and ETO in B16-F10 cells. (**b**,**d**) was quantitative analysis of (**a**,**c**), respectively, which was obtained by using Image J software 1.52a (National Institutes of Health, Bethesda, MD, USA). The analysis was repeated in SK-MEL-2-Luc cells. Western blot data of (**e**) 24 h and (**g**) 72 h was shown, and the corresponding quantitative analysis was shown in (**f**,**h**), respectively. GAPDH was used as the control of sample loading. Relative intensities of Ku70/KU70 to GAPDH expressions were normalized with the control. The data are shown as means ± SEM of independent experiments in triplicate. The drug dosages were referred to IC_50_ values as shown in Figure 2 and Table 1.

Furthermore, we found that the expression levels of Rad51/RAD51, a key protein that plays an essential role in the HR pathway, were remarkably dominated by ETO administration in both cells under 24 h-simultaneous and sequential combined treatments (Figure 8a,b,e,f). We also found that Rad51 expression of B16-F10 cells decreased to 60% under simultaneous treatment but increased to 1.90-fold under VPA→ETO sequential treatment for 72 h compared to the control (Figure 8c,d). In comparison, RAD51 in SK-MEL-2-Luc cells was down-regulated to around 10% under VPA single treatment, simultaneous, and ETO→VPA sequential treatments for 72 h (Figure 8g,h). On the other hand, the expression levels of Ku70/KU70 and Ku80/KU80, which constitute the Ku/KU heterodimer involved in the NHEJ pathway, exhibited no significant change in all experimental groups compared to the control (Figure 9 and Figure 10). These data implied that there was no difference in NHEJ activity among all treatment schemes.

Taken together, the expression profile analyses of HR or NHEJ marker proteins suggested that the DNA DSBs induced by VPA→ETO sequential treatment might be mainly repaired via the HR pathway.

### 2.5. Different Sequential Orders of VPA and ETO Administration Might Influence the Cell Cycle Regulation

We next evaluated whether the different sequential orders of VPA and ETO administration influence the cell cycle regulation in B16-F10 and SK-MEL-2-Luc cells.

**Figure 11:**
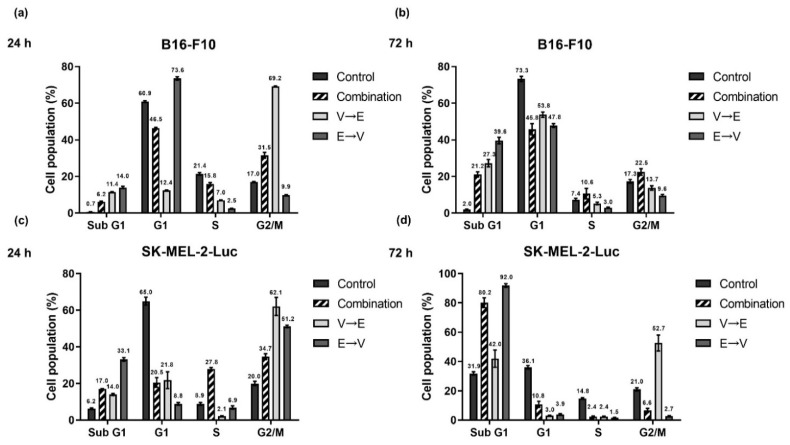
Cell cycle analysis of simultaneous and sequential combined treatments of VPA and ETO in melanoma cells. Cell cycle progression was determined by flow cytometry with propidium iodide (PI) staining of DNA. (**a**) The quantitative analysis of cell cycle population of B16-F10 cells under 24 h single, simultaneous, and sequential combined treatments of VPA and ETO, and under (**b**) 72 h of the specified treatment scheme was shown. The analysis was repeated in SK-MEL-2-Luc cells as shown in (**c**,**d**). The data are shown as means ± SEM of independent experiments in triplicate. The drug dosages were referred to IC_50_ values as shown in Figure 2 and Table 1.

We analyzed the cell populations in each cell cycle phase under simultaneous or sequential combined treatments of VPA and ETO (Figure 11). We found that the cell populations in the sub-G1 phase significantly increased under 72 h ETO→VPA sequential treatment compared to the control (Figure 11b,d). Under VPA→ETO sequential treatment, the B16-F10 cell populations were arrest in the G2/M phase for 24 h (Figure 11a), while the SK-MEL-2-Luc cell populations were arrest in G2/M (Figure 11c,d). These results implied that VPA→ETO sequential treatment might induce G2/M phase arrest while ETO→VPA sequential treatment might result in a larger population of sub-G1 phase in both cell lines.

**Figure 12:**
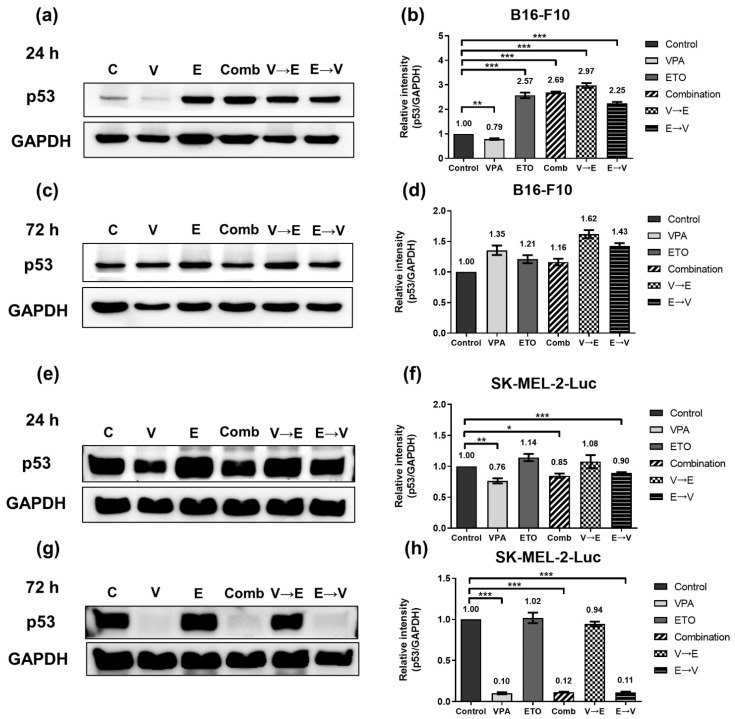
Expression of cell cycle regulator p53, under single, simultaneous, and sequential combined treatments of VPA and ETO in melanoma cells. Western blot analysis illustrated the p53 protein expression after (**a**) 24 h (**c**) 72 h single, simultaneous, and sequential combined treatments of VPA and ETO in B16-F10 cells. (**b**,**d**) was quantitative analysis of (**a**,**c**), respectively, which was obtained by using Image J software 1.52a (National Institutes of Health, Bethesda, MD, USA). The analysis was repeated in SK-MEL-2-Luc cells. Western blot data of (**e**) 24 h and (**g**) 72 h was shown, and the corresponding quantitative analysis was shown in (**f**,**h**), respectively. GAPDH was used as the control of sample loading. Relative intensities of p53 to GAPDH expressions were normalized with the control. The data are shown as means ± SEM of independent experiments in triplicate. (* *p* < 0.05; ** *p* < 0.01; *** *p* < 0.001). The drug dosages were referred to IC_50_ values as shown in Figure 2 and Table 1.

**Figure 13:**
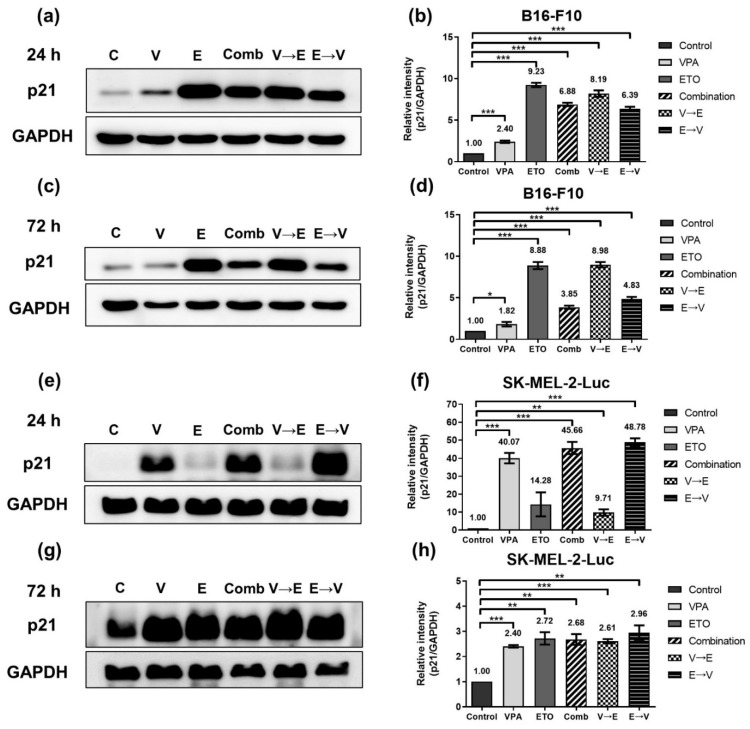
Expression of cell cycle regulator p21, under single, simultaneous, and sequential combined treatments of VPA and ETO in melanoma cells. Western blot analysis illustrated the p21 protein expression after (**a**) 24 h (**c**) 72 h single, simultaneous, and sequential combined treatments of VPA and ETO in B16-F10 cells. (**b**,**d**) was quantitative analysis of (**a**,**c**), respectively, which was obtained by using Image J software 1.52a (National Institutes of Health, Bethesda, MD, USA). The analysis was repeated in SK-MEL-2-Luc cells. Western blot data of (**e**) 24 h and (**g**) 72 h was shown, and the corresponding quantitative analysis was shown in (**f**,**h**), respectively. GAPDH was used as the control of sample loading. Relative intensities of p21 to GAPDH expressions were normalized with the control. The data are shown as means ± SEM of independent experiments in triplicate. (* *p* < 0.05; ** *p* < 0.01; *** *p* < 0.001). The drug dosages were referred to IC_50_ values as shown in Figure 2 and Table 1.

G2/M checkpoint is the most critical checkpoint throughout cell cycle progression. After receiving the signal of DNA damage, p53 will up-regulate p21 to trigger G2/M phase arrest [19]. We, therefore, investigated the expression levels of p53 and p21 in both cell lines (Figure 12 and Figure 13). These data implied ETO dominated the p53 up-regulation in B16-F10 cells within 24 h. However, there was no significant difference in the expression levels of p53 between B16-F10 cells under all 72 h drug administration conditions. (Figure 12c,d).

On the other hand, the protein expression of p53 in SK-MEL-2-Luc cells slightly reduced under VPA single treatment, simultaneous, and ETO→VPA sequential treatments (Figure 12e,f). Notably, the expression levels of p53 in SK-MEL-2-Luc cells significantly reduced under the same conditions (Figure 12g,h).

As shown in Figure 13a–d, the expression levels of p21 significantly increased in B16-F10 cells with ETO treatment, which meant ETO dominated the initial up-regulation of p21. However, as shown in Figure 13c,d, the expression levels of p21 reduced from 24 to 72 h under simultaneous (i.e., 6.88- to 3.85-fold) and ETO→VPA sequential treatment (i.e., 6.39- to 4.83-fold). These results indicated that VPA might counteract the up-regulation of p21 by ETO in this treatment scheme. The counteracting effect of ETO and VPA on p21 expression was also observed in SK-MEL-2-Luc cells (Figure 13e–h).

### 2.6. ETO Pretreated Sequential Combined Treatment Augmented Apoptosis via Caspase-3 Up-Regulation

Lastly, we investigated whether apoptosis was involved in the synergistic inhibitory effects under simultaneous or sequential combined treatments of VPA and ETO.

**Figure 14:**
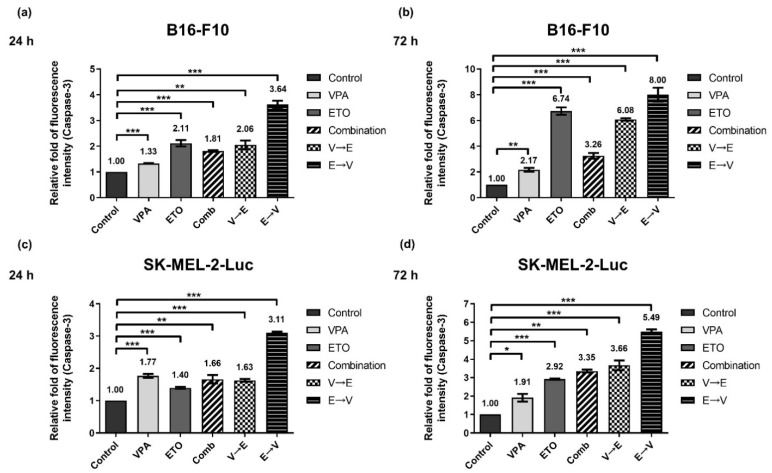
Caspase-3 activity analysis of single, simultaneous, and sequential combined treatments of VPA and ETO in melanoma cells. Caspase-3 activity was evaluated by flow cytometry with phycoerythrin (PE) staining. (**a**) The quantitative analysis of the caspase-3 activity of B16-F10 cells under 24 h single, simultaneous, and sequential combined treatments of VPA and ETO, and (**b**) 72 h of the specified treatment scheme was shown. The analysis was also repeated in SK-MEL-2-Luc cells, as shown in (**c**,**d**). The data are shown as means ± SEM of independent experiments in triplicate. (* *p* < 0.05; ** *p* < 0.01; *** *p* < 0.001). The drug dosages were referred to IC_50_ values as shown in Figure 2 and Table 1.

As shown in Figure 14, we observed elevated activities of caspase-3 in all experimental groups for 24 and 72 h. Notably, the caspase-3 activities under ETO→VPA sequential treatment increased from 24 to 72 h in B16-F10 (i.e., 3.64- to 8.00-fold) and SK-MEL-2-Luc cells (i.e., 3.11- to 5.49-fold). This finding indicated that B16-F10 and SK-MEL-2-Luc cells under ETO→VPA sequential treatment exhibited the highest apoptosis tendency in cell fate decisions. Together, the results suggested that ETO→VPA sequential treatment contributed to the enhanced apoptosis, which might exert the synergistic inhibitory effect on B16-F10 and SK-MEL-2-Luc cells. 

## 3. Discussion

In this study, we demonstrated the synergistic inhibitory effects on murine melanoma cell line B16-F10 and human melanoma cell line SK-MEL-2-Luc under simultaneous and sequential combined treatments of VPA and ETO. However, when drugs were given in different tandem orders, we observed drug antagonism under the VPA→ETO sequential treatment. Our finding suggested that sequential orders of VPA and ETO administration contributed to different cellular responses.

Based on the synergy analysis, we found two schemes: (1) simultaneous combined treatment of VPA and ETO, and (2) ETO→VPA sequential combined treatment, could display synergistic inhibitory effects on B16-F10 and SK-MEL-2-Luc cells (Figure 3, Figure 4 and Figure 5 and Supplementary Materials Table S2). Our findings further revealed that synergism might only exist within a limited range of combined drug concentrations. These data also suggested that the range of effective dose for exhibiting synergism in SK-MEL-2-Luc cells might be broader than the corresponding range in B16-F10 cells.

On the other hand, we pretreated the first drug for 24 h in the sequential combined treatments following the experimental design of Valentini et al. [7]. M14 melanoma cells have been reported to demonstrate similar levels of cell growth inhibition under 24 and 48 h single treatments [11]. Thus, we might obtain a higher or similar level of cell inhibition in melanoma cells with 48 h pretreatment, while 12 h pretreatment might result in less efficacy in terms of potential temporal effects. In addition, we administrated VPA once in the ETO→VPA sequential combined treatment since VPA is stable in solution under 40 °C [20]. We, therefore, considered there would be a negligible difference in efficacy when VPA is given at regular intervals.

DNA DSB disrupts the genomic integrity and the fidelity of DNA replication in cells. There are two main DNA repair pathways in eukaryotic cells, HR and NHEJ, responsible for repairing DNA DSBs. It is generally believed that HR is a more accurate and error-free DNA repair pathway, while NHEJ results in more imprecise and error-prone repair [21]. Our analysis showed that ETO dominated the induction of γH2AX (Figure 6). We also found that DNA DSBs induced by VPA→ETO sequential treatment was predominantly repaired by the HR pathway (Figure 7 and Figure 8). Rad51/RAD51 is the central player in the HR pathway for repairing DNA double-strand breaks. Our findings were consistent with the previous study that showed VPA could down-regulate both RNA and protein expression levels of RAD51 in human melanoma cells [22]. Therefore, the decreased expression of Rad51/RAD51 caused by VPA pretreatment might interfere with cellular responses, enhancing the Rad51/RAD51 expression following ETO administration.

The G2/M checkpoint prevents cells from entering mitosis when they harbor unrepaired DNA lesions during previous S or G1 phases. The accumulation of cells in the G2/M phase indicates that the G2/M checkpoint is activated by DNA damage detection [23]. In this study, we found that more cells were arrested at the G2/M phase in melanoma cells after VPA→ETO sequential treatment, implying the effect on cell cycle checkpoint control (Figure 11).

HR is known to be activated in the late S and G2/M phases only, as it requires an integrated homologous DNA as the template to enact the DNA repair [21]. Coincidentally, our data showed that the expression levels of HR-related proteins, such as Chk2/CHK2 and Rad51/RAD51, were increased or maintained at similar expression levels under VPA→ETO sequential treatment compared to the control (Figure 7 and Figure 8). The resulting augmented HR activity could contribute to cell survival, leading to drug antagonism in the cells under the VPA pretreatment followed by successive ETO treatment. Our data suggested that G2/M phase arrest and the enhanced HR activity might contribute to an antagonistic effect on melanoma cells under VPA→ETO sequential treatment.

On the other hand, the concurrent administration of VPA might enhance the extent of ETO-induced DNA DSBs under simultaneous combined treatment, which was evident in the increased expression levels of γH2AX (Figure 6). At the same time, we observed that the HR repair activities might be compromised in this scheme (Figure 7 and Figure 8). Taken together, the augmented DNA DSBs and the compromised HR activity might contribute to the synergistic inhibitory effect under VPA-ETO simultaneous combined treatment.

When DNA lesions are too severe to repair, cells will exit the normal cell cycle and fall into the sub-G1 phase, which corresponds to pro-apoptotic cells [24]. Our data indicated that the ETO→VPA sequential treatment scheme could significantly induce apoptosis. Our data further revealed that the pretreatment of VPA might attenuate the level of ETO-induced apoptosis in the cells. In contrast, with ETO pretreatment, VPA could push the cells toward apoptosis, which resulted in boosted apoptosis and contributed to the synergistic inhibitory effect under the ETO→VPA sequential treatment scheme (Figure 14). Our findings highlighted how the cellular outcomes could be altered in multiple aspects, including reaction time, intensity of reaction, and duration of reaction in cells under different sequential orders of drug administration. These results highlighted the importance and the need for pre-clinical study of drug administration schemes to avoid ineffective treatment during cancer combination therapy.

In a previous phase I/II clinical trial [14], VPA has been used in combination with chemo-immunotherapy on patients suffering from advanced melanoma. However, the clinical outcomes of VPA and chemo-immunotherapy combination were not superior to standard therapy, and more adverse effects were observed in these advanced melanoma patients during the trial. It is noteworthy that the patients who participated in this clinical trial received a 6-week VPA pretreatment before the concurrent treatment of VPA and chemo-immunotherapy [14]. Given our findings, the 6-week VPA pretreatment might have contributed to the antagonistic effect on melanoma cell inhibition.

In conclusion, our present study demonstrated the significance of how sequential orders and effective doses of drug administration in combination therapy result in different clinical outcomes. Therefore, multi-drug treatment in the proper dose and sequential order shall be carefully analyzed before clinical applications.

## 4. Materials and Methods

### 4.1. Cell Lines and Cell Culture

The murine melanoma cell line B16-F10 (ATCC^®^ CRL-6475™, American Type Culture Collection, Manassas, VA, USA) and the human melanoma cell line SK-MEL-2-Luc (ATCC^®^ HTB-68™, American Type Culture Collection, Manassas, VA, USA) were used. Both B16-F10 and SK-MEL-2-Luc cells were cultured in Dulbecco’s Modified Eagle’s Medium (DMEM; Gibco, New York, NY, USA) that contained 10% fetal bovine serum (FBS; Gibco, New York, NY, USA), 100 unit/mL streptomycin (Gibco, New York, NY, USA), 100 μg/mL penicillin (Gibco, New York, NY, USA), 0.25 μg/mL fungizone (Gibco, New York, NY, USA) and 3.7 g sodium bicarbonate (Sigma-Aldrich, St. Louis, MO, USA) at 37 °C in ambient air with 5% CO_2_.

### 4.2. Drugs

Valproic acid (VPA; Sigma-Aldrich, St. Louis, MO, USA; Cat. #P4543-10G) was dissolved in phosphate-buffered saline (PBS; Hyclone Laboratories Inc., Logan, UT, USA; Cat. #SH30256.02) into 300 mM and stored at −20 °C. Etoposide (ETO; Selleckchem, Houston, TX, USA; Cat. #S1225) was dissolved in dimethyl sulfoxide (DMSO; Sigma-Aldrich, St. Louis, MO, USA; Cat. # D2650-100 mL) into 100 mM and stored at −20 °C. Both VPA and ETO were diluted into desired working concentrations in culture medium prior to experiments.

### 4.3. Cell Viability Assay and IC_50_ Determination

Cells were seeded in 96-well plates at a density of 1000 cells/well and incubated 24 h at 37 °C before drug administration. All experimental groups were categorized into three conditions: (1) single treatments (i.e., VPA or ETO), (2) simultaneous combined treatment of VPA and ETO, and (3) sequential combined treatment (i.e., V→E or E→V sequential treatment). In the groups of single agents and simultaneous combined treatment, the cells were treated with single drugs or a concurrent combination of VPA and ETO for 72 h. In the groups of sequential combined treatment, the cells were pretreated with single drugs for 24 h then treated with the other single drugs for 72 h. After the drug administration, a 10 μL Cell Counting Kit-8 cell proliferation reagent (CCK-8; Dojindo, Kumamoto, Japan) was added to each well of 96-well plates then incubated at 37 °C and 5% CO_2_ for 2 h. The optical densities were measured at 450 nm wavelength. The IC_50_ is the concentration of a drug that is required for 50% cell inhibition in vitro, which can be calculated by GraphPad Prism 8 (GraphPad Software, San Diego, CA, USA) [15]. The obtained IC_50_ values of VPA and ETO were used in subsequent experiments.

### 4.4. Analysis of Combination Index

The synergisms of VPA and ETO under different sequential orders were evaluated by combination index methods described by Chou and Talalay [16,17]. The cell viability data were converted into a fraction of growth affected (Fa) under single treatments, simultaneous combined treatment, and sequential combined treatments. Next, CompuSyn calculation software, 2005 Edition (ComboSyn, Paramus, NJ, USA) calculated the combination index (CI) values based on the Fa values uploaded. The range of CI values are defined as CI > 1, CI = 1, and CI < 1, indicating the antagonistic, additive, and synergistic effects, respectively. For data visualization, Fa-CI plots and isobolograms were generated by CompuSyn. The Fa-CI plot represents the interaction of two drugs quantitatively. A specific Fa value or effective dose (ED) value of corresponding dose concentration is plotted on the isobologram, which could be used for discriminating therapeutic effects from synergistic and antagonistic effects.

### 4.5. Western Blot Analysis

The experimental process followed the protocols described by Chen et al. with modification [25]. B16-F10 and SK-MEL-2-Luc cells were treated with a single treatment of VPA or ETO, simultaneous and sequential combined treatments of VPA and ETO. The chemiluminescent signals of individual protein were recorded on films (Fujifilm, Tokyo, Japan) for analysis. Some membranes were intensified for their chemiluminescent signals of individual protein by Amersham ECL Prime Western Blotting Detection Reagent (GE Healthcare, Chicago, IL, USA) and then recorded the signals by Luminescence/Fluorescence Imaging System (GE Healthcare, Chicago, IL, USA; Life Sciences ImageQuant; LAS 4000). The anti-Ku80/KU80 (Cat. #2180), Ku70/KU70 (Cat. #4588), Chk2/CHK2 (Cat. #2662), p53 (Cat. #2524 for B16-F10 cells; Cat. #9282 for SK-MEL-2-Luc cells), Rad51/RAD51 (Cat. #8875), p21 (Cat. #64016 for B16-F10 cells; Cat. #2947 for SK-MEL-2-Luc cells), and γH2AX (Cat. #9718) antibodies were obtained from Cell Signaling Technology (Danvers, MA, USA). The GAPDH (Cat. #GTX627408) antibody was obtained from GeneTex (Irvine, CA, USA).

### 4.6. Cell Cycle and Caspase-3 Apoptosis Analysis

The experimental process followed the flow cytometry protocols described by Chen et al. with modification [25]. B16-F10 and SK-MEL-2-Luc cells were treated with a single treatment of VPA or ETO, simultaneous and sequential combined treatments of VPA and ETO.

### 4.7. Statistics

Data were processed by GraphPad Prism 8 (GraphPad Software, San Diego, CA, USA) and expressed as the means ± standard error of the mean (SEM). Statistical significance was evaluated by a two-tailed Student’s *t*-test (*p*-value < 0.05) with SigmaPlot 12.0 (Systat Software, Chicago, IL, USA).

## Figures and Tables

**Table 1:** IC_50_ values of valproic acid (VPA) or etoposide (ETO) single treatment on B16-F10 and SK-MEL-2-Luc cells.

**Cell Line**	**B16-F10**	
Single-drug treatment	Valproic acid (VPA)	Etoposide (ETO)
IC_50_ value	8.72	6.25
Unit	mM	μM
**Cell Line**	**SK-MEL-2-Luc**	
Single-drug treatment	Valproic acid (VPA)	Etoposide (ETO)
IC_50_ value	5.34	5.58
Unit	mM	μM

## Data Availability

Not applicable.

## Supplementary Materials

The following are available online at https://www.mdpi.com/article/10.3390/ijms221810029/s1.

## Abbreviations

Caspase-3: Cysteine-aspartic protease-3; Chk2/CHK2: Checkpoint kinase 2; CI: Combination index; DSB: Double-strand break; ED: Effective dose; ED_50,75,90_: 50%, 75%, 90% effective dose; ETO: Etoposide; Fa: Fraction of affected cells; GAPDH: Glyceraldehyde-3-phosphate dehydrogenase; HDAC: Histone deacetylase; HR: Homologous recombination; IC_50_: Half maximal inhibitory concentration; NHEJ: Non-homologous end joining; PE: Phycoerythrin; PI: Propidium iodide; VPA: Valproic acid.
