# Supplementary material for: Synergistic Effect of Simultaneous versus Sequential Combined Treatment of Histone Deacetylase Inhibitor Valproic Acid with Etoposide on Melanoma Cells
Source: Int J Mol Sci. 2021 Sep 17;22(18):10029. doi: 10.3390/ijms221810029 (PMC8467070; doi:10.3390/ijms221810029)
Supplement: Supplementary file 1 [file ijms-22-10029-s001.zip › ijms-1331008-supplementary.pdf]

Supplementary Information

**Range of CI**	**Graded symbol**	**Drug effect**
<0.1	+ + + + +	Very strong synergism
0.1-0.3	+ + + +	Strong synergism
0.3-0.7	+ + +	Synergism
0.7-0.85	+ +	Moderate synergism
0.85-0.90	+	Slight synergism
0.90-1.10	±	Nearly additive
1.10-1.20	–	Slight antagonism
1.20-1.45	– –	Moderate antagonism
1.45-3.3	– – –	Antagonism
3.3-10	– – – –	Strong antagonism
>10	– – – – –	Very strong antagonism

Supplementary Table S1. Categorization of combination index (CI) of drug combination to corresponding drug effect and grading

This table is adapted from the paper published by Chou [17].

**Cell line**	**B16-F10**		
**Drug sequential order of combination treatment**	**CI values and gradings**		
	**ED_{50}**	**ED_{75}**	**ED_{90}**
**Combination** (Simultaneous combined treatment of VPA and ETO)	0.57<br>+ + +	0.73<br>+ +	1.42<br>– –
**V→E** (Sequential combined treatment that pre-treated with VPA)	1.77<br>– – –	3.52<br>– – – –	10.58<br>– – – – –
**E→V** (Sequential combined treatment that pre-treated with ETO)	0.21<br>+ + + +	1.15<br>–	9.55<br>– – – –

**Cell line**	**SK-MEL-2-Luc**		
**Drug sequential order of combination treatment**	**CI values and gradings**		
	**ED_{50}**	**ED_{75}**	**ED_{90}**
**Combination** (Simultaneous combined treatment of VPA and ETO)	0.30<br>+ + +	0.39<br>+ + +	0.68<br>+ + +
**V→E** (Sequential combined treatment that pre-treated with VPA)	1.17<br>–	11.38<br>– – – – –	147.31<br>– – – – –
**E→V** (Sequential combined treatment that pre-treated with ETO)	0.32<br>+ + +	0.43<br>+ + +	0.77<br>+ +

Supplementary Table S2. Combination index (CI) values and synergism/antagonism grading of simultaneous and sequential combined treatments of VPA and ETO on B16-F10 and SK-MEL-2-Luc cells
